# Primary Mediastinal Germ Cell Tumors: A Thorough Literature Review

**DOI:** 10.3390/biomedicines11020487

**Published:** 2023-02-08

**Authors:** Guliz Ozgun, Lucia Nappi

**Affiliations:** Department of Medical Oncology, British Columbia Cancer Agency Vancouver Center, Vancouver, BC V5Z 4E6, Canada

**Keywords:** mediastinal germ cell tumors (MGCTs), clinical features, poor prognosis, treatment, molecular features

## Abstract

Primary mediastinal germ cell tumors (PMGCTs) are a rare type of cancer affecting young adults. They have different molecular and clinical features compared to testicular germ cell tumors. Non-seminoma PMGCTs have the shortest 5-year overall survival and the poorest prognosis among all of the germ cell tumor presentations, while seminomas share the same survival and prognosis as their testicular counterparts. There is an unmet need for better treatment options for patients with non-seminoma PMGCTs in both first-line and salvage therapy, as the available options are associated with underwhelming outcomes. Identifying biological and genetic factors to predict treatment responses would be helpful in improving the survival of these patients.

## 1. Introduction

GCTs mainly originate from the gonads. However, 2–5% of cases arise in the midline structures, with the mediastinum being the most common site in adults aged between 25 and 35. PMGCTs account for 15–20% of all anterior mediastinal tumors [1,2]. Non-seminoma is more frequent than seminoma in PMGCTs representing 60–70% of cases. Mature teratoma is the most frequent histology among non-seminoma PMGCTs and is usually managed with surgery. Conversely, immature teratomas are rarer aggressive tumors with poorer outcomes [1,3,4]. Primary mediastinal seminomas have excellent cure rates similar to their testicular counterparts, with a 5-year survival rate exceeding 90% [5]. Contrarily, lower cisplatin sensitivity and scarcity of alternative treatment options in the first and later lines make treatment challenging in non-seminoma PMGCTs. Biomarker and actionable target discovery studies are ongoing for a better treatment approach. This paper presents a comprehensive review of mediastinal germ cell tumors literature.

## 2. Methodology

Relevant studies published between 1976 and 2022 were identified via PubMed and Google Scholar search using different combinations of “mediastinal germ cell tumors (MGCTs)”, “clinical features”, “prognostic factors”, “treatment”, and “molecular features.” Additional papers were identified by reviewing the reference lists of relevant publications. Publications with relatively low reliability, written in another language than English, and studies conducted on animals were excluded. Data were extracted based on their relevance to the topic.

## 3. Clinical Features

PMGCTs arise more frequently from the anterior mediastinum and rarely from the posterior, middle, or superior mediastinum. PMGCTs account for 15–20% of mediastinal tumors in young men aged between 25 and 35 [1]. Dyspnea, cough, chest pain, and weight loss are typical symptoms at the time of diagnosis. The other accompanying symptoms include hemoptysis, superior vena cava (SVC) syndrome, recurrent laryngeal nerve palsy, fever, night sweats, nausea, and gynecomastia. PMGCTs primarily metastasize to the mediastinal lymph nodes. However, they can also metastasize to the lungs, liver, bone, retroperitoneum, central nervous system, and heart [5]. As extra-mediastinal metastasis is related to poorer prognosis, patients should be staged with appropriate diagnostic tests that include whole-body CT scans.

In differential diagnosis, thymic diseases, thyroid goiter, NUT carcinoma (a rare SALL4- and AFP-expressing poorly differentiated squamous cell cancer)*,* metastatic melanoma, sarcomas (the SMARCA4-deficient subtype often expresses *SOX2*, *SALL4*, and vimentin), lymphomas (which can be identified with CD45a and B/T cell markers), and metastatic carcinoma to the mediastinum should be considered. Moreover, dissemination following the course of the thoracic duct can be associated with a testicular or a retroperitoneal primary. Therefore, additional workup should be carried out to evaluate testicular primary.

Although the histological and pathological features of PMGCTs are not different from gonadal GCTs, the clinical presentation, symptomatology, and prognosis may differ from their gonadal counterparts. Non-seminoma PMGCTs have a poor prognosis, with a 5-year overall survival (OS) of 40%–50% despite chemotherapy and surgery (Table 1). Independent prognostic factors associated with shorter survival in non-seminoma PMGCTs are non-pulmonary visceral metastases and elevated β-HCG. Conversely, seminoma PMGCTs have an excellent prognosis with an OS exceeding 90% using the current curative treatment modalities [5,6,7,8].

## 4. Tumor Markers

While non-seminoma PMGCTs are associated with high levels of LDH, AFP, and βHCG, around 20% of seminomas are characterized by elevated βHCG and LDH [12]. AFP is elevated in embryonal carcinoma, yolk sac tumors, and teratoma; βHCG is elevated in seminoma, choriocarcinoma, and embryonal carcinoma. The clinical utility of LDH is limited by its lack of specificity. High and increasing levels of AFP in a seminoma patient implies ruling out non-seminoma components since these patients should be considered and treated as non-seminoma. Novel circulating micro-RNAs, including miR371a-3p, have been described in GCTs. Their sensitivity and specificity are remarkably higher than the classic serum tumor markers AFP, βHCG, and LDH, which have a combined sensitivity of 50%. Although mediastinal-specific GCT data about the expression and detection of these miRNAs in the peripheral blood are lacking, it appears that miR371a-3p is also detectable in the plasma of PMGCTs harboring seminoma or non-seminoma viable active malignancies while is not detectable in patients with mediastinal teratoma [13].

## 5. Histopathology and Embryogenesis

Interrupted migration of progenitor germ cells during embryogenesis, burnt-out primary (healed testis primary at extragonadal GCT diagnosis), and reverse migration of transformed germ cells from testes are the proposed mechanisms so far for developing extragonadal GCTs [14,15,16].

PMGCTs are classified as non-seminoma, including teratoma (mature, immature, and teratoma with somatic malignancies), yolk sac tumors, choriocarcinoma, embryonal carcinoma, mixed germ cell tumors, germ cell tumors with associated hematological malignancy (WHO 2021 classification), and seminoma [17]. While teratoma (58%) and yolk sac tumors (42%) make up a substantial part of prepubertal extra-gonadal GCTs, the most common histological subtype in adults is mature teratoma [18]. GCTs are also classified into five groups regardless of their primary site, defined by chromosomal changes and developmental potential. Type I GCTs comprise infantile teratomas and yolk sac tumors with the loss of chromosomes 1p, 4, and 6q and the gain of chromosomes 1q, 12(p13), and 20q. Type II GCTs include seminomas and non-seminomas in adolescent/adult men and typically have a gain of chromosomes 7, 8, 12p, 21, and X and a loss of chromosomes 1p, 11, 13, and 18 [19].

Primary mediastinal choriocarcinoma is rare and most patients have hematogenous dissemination at diagnosis. Therefore, it has a poorer prognosis when compared to other histologic subtypes [20]. Embryonal carcinoma cells are considered malignant variants of embryonal stem cells and they share biochemical and morphologic similarities. A yolk sac tumor usually includes malignant endodermal and extraembryonic mesenchymal cells, which are more commonly observed in children. Yolk sac tumors and embryonal carcinoma are associated with poor prognosis.

Teratomas arise from the three germinal layers and have the potential to differentiate into any tissue in the body. Immaturity and quantification of the neuroepithelial component are used for teratoma grading. Grade 1 is defined as tumors with some degree of immaturity, but neuroepithelium presence is limited to a maximum of one focus. Grade 3 is defined as the existence of a significant immaturity and neuroepithelium, with neuroepithelial components in ≥4 fields within individual parts. Grade 2 stands between grades 1 and 3 [21]. Unlike testicular GCTs (TGCTs), mature and immature teratoma differentiation is critical for MGCT patients’ management since immature teratomas have the potential for aggressive behavior. Mature teratoma is the most common form of teratoma in PMGCTs (63%), while immature teratoma is diagnosed in about 4% of patients. Teratoma with sarcoma, other malignant germ cell elements, or carcinoma is observed in about 33% of cases [22].

SALL4, PLAP (placental alkaline phosphatase), organic cation transporter (OCT) 3–4, NANOG, c-kit (CD117), CD30, EMA (epithelial membrane antigen), cytokeratins, FP, -HCG, and glycipan-3 are the validated immunohistochemical markers for the pathological diagnosis confirmation of GCTs. Since there are often variable and focal stainings depending on the tumor phenotype, immunohistochemical antibodies should be used to detect the proteins.

Seminomas are positive for PLAP, OCT-4, c-kit, and SALL-4 and negative for CD30 and cytokeratins. Conversely, embryonal carcinoma is almost always positive for cytokeratins. EMA, CD30, OCT-4, and SALL-4 can also be positive while PLAP is positive in about 50% of cases. Likewise, yolk sac tumors are positive for cytokeratins and SALL-4 but negative for CD30 and c-kit (Table 2). Leucocyte common antigen (LCA; CD45) and desmin/vimentin are used for the differential diagnosis of lymphomas and sarcomas, respectively. Non-germ cell malignant transformation occurs more frequently in teratoma PMGCTs than in gonadal or primary retroperitoneal GCTs and consists of sarcoma and adenocarcinoma differentiation [23].

Post-chemotherapy residual disease is characterized by 40–50% fibrosis and necrosis and mixed inflammatory aggregates, 10–20% viable GCTs, and 30–40% teratoma [24,25]. A pathology report should contain the ratio of viable non-teratoma GCTs since treatment response is one of the most critical factors in predicting long-term outcomes. Viable tumor cells below 10% represents a good prognostic factor [26]. Overall, sampling should be extensive to better appreciate the residual tumor, if present.

## 6. Genomic Features

**Figure 1:**
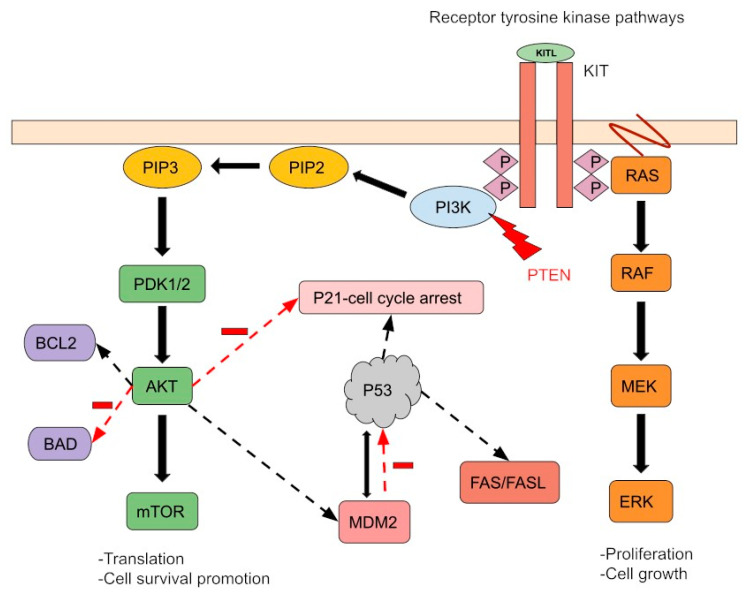
A simple schema of molecular aberrations in PMGTs. The most common mutations described in PMGCTs are *TP53* (46%), *c-KIT* (18%), *KRAS* (18%), *PTEN* (11%), *NRAS* (4%), and *PIK3CA* (4%). *K-RAS* and *N-RAS* mutations cause Ras-Raf-MEK-ERK pathway activation regardless of receptor activity. The *PTEN* tumor suppressor gene controls cell growth and migration. Its mutation activates the PI3K pathway, leading to impaired mitotic arrest and cell survival promotion. MDM2 and P53 regulate each other through feedback mechanisms. *P53* mutation and/or MDM2-induced ubiquitination and disruption of P53 inhibit apoptosis. Finally, the *c-KIT* gain-of-function mutation and/or *KIT* overexpression activates downstream signaling by increasing receptor tyrosine kinase activity. Furthermore, these pathways can interact and co-regulate downstream processes, promoting cell survival and proliferation.

The i(12p) is a cytogenetic aberration identified in approximately 80% PMGCTs regardless of the histological subtype [27]. In suspicious cases, which have lost their typical GCT appearance, documenting i(12p) persistence is a helpful tool for molecular diagnosis. Therefore, identification of i(12p) in a mediastinal mass specimen (usually with fluorescence in situ hybridization) could confirm a diagnosis of PMGCTs [28]. Several genes including *MED21*, *Sox5*, *DAD-R*, *BCAT1*, *KRAS*, *Cyclin D2*, *FGF6*, *ATF7IP*, *GDF3*, *LRP6*, *Wnt5B*, *FGF23*, *Nanog*, and *Dppa3* are located on the short arm of chromosome 12 and could play a role in GCT development [29]. However, the exact molecular mechanisms leading to GCT initiation and progression remain unclear. Other chromosomal abnormalities involve chromosomes 1p, 1q, and 6q and the sex chromosomes. Compared to testicular GCTs, PMGCTs have a higher tumor mutational burden and specific pathogenic oncogene alterations. The most common mutations described in PMGCTs are: *TP53* (46%), *c-KIT* (18%), *KRAS* (18%), *PTEN* (11%), *NRAS* (4%), and *PIK3CA* (4%) (Figure 1). These alterations are more common in non-seminoma PMGCTs than in seminomas and non-seminoma TGCTs. Few studies have correlated *TP53* mutations and *MDM2* alterations to cisplatin resistance in GCTs. While TP53 mutations are primarily found in mediastinal GCTs, *MDM2* amplifications are mainly found in the testis [30]. In a retrospective analysis, *TP53* alterations were detected in 16.3% (17/104) of patients with cisplatin-resistant GCTs. Of note, *TP53* mutations were observed in 72.2% (13/18) of the non-seminoma PMGCTs samples analyzed in that study [31]. In a recent multi-institutional study focused on PMGCTs, *TP53* genomic alterations were described in 56% of non-seminoma tumors and were associated with significantly shorter OS than in patients with wild-type *TP53* PMGCTs, suggesting a distinct genomic background in patients with PMGCTs which could explain the poor prognosis of this patient population [32]. *TP53* alterations are even more frequent in PMGCTs associated with hematologic malignancies where they have been observed in 91% of the patients [33].

## 7. Association with Other Diseases and Malignancies

Non-seminoma PMGCTs can be associated with chromosomopathies and hematologic malignancies (HMs). Klinefelter’s syndrome (KS; 47, XXY) is the most common chromosomal disease associated with non-seminoma PMGCTs. According to the Children’s Oncology Group, approximately one-third of patients with PMGCT have KS [34]. Therefore, PMGCT patients should be screened for KS. There are few case reports describing PMGCT in neurofibromatosis type 1 patients [35].

HMs are seen in 2–3% of non-seminoma PMGCT patients, either simultaneously (31%) or after initial diagnosis (46%) and are associated with a very poor prognosis and OS of less than 2 years. The most common HM in PMGCTs patients is acute megakaryoblastic leukemia (AML-M7). Other HMs include other AMLs, chronic myeloid leukemia, acute lymphoblastic leukemia, myelodysplasia, malignant mastocytosis, essential thrombocytopenia, and malignant histiocytosis [36,37,38]. i(12p) is detected in about 47% of secondary HM cells. Other genomic alterations including *TP53*, *KRAS*, and *PTEN* can also be present, suggesting that these cells are derived from a mutual descent [36,39]. Therefore, hematologic malignancies secondary to PMGCTs genetically resemble PMGCTs rather than primary HMs. There are no guidelines for non-seminoma PMGCT-associated hematologic malignancy treatment. The case reports/series show a limited response rate to current hematologic chemotherapy protocols. Therefore, treating this disease as AML with poor prognostic features is reasonable. They should also be promptly considered for allogeneic hematopoietic stem cell transplantation, even if definitive GCT treatment is delayed [29].

Etoposide-related HMs should be considered when a PMGCT patient is diagnosed with hematologic malignancy. Therapy-related diseases would emerge later, approximately 25–60 months after chemotherapy treatment. Furthermore, they possibly have etoposide-related translocations such as 11q23 and usually are negative for i(12p).

Somatic transformation occurs in 1–2% of male GCTs of which 25–30% are PMGCTs. The most common somatic transformations include carcinoma (adenocarcinoma, adenoid cystic carcinoma, and high-grade neuroendocrine carcinoma), sarcoma (angiosarcoma, neurogenic sarcoma, rhabdomyosarcoma, and high-grade sarcoma, not otherwise specified), primitive neuroectodermal tumors, and Wilms tumors, which are related to poor prognosis [40]. Conversely, somatic transformation in a metastatic rather than primary TGCT lesion is associated with an increased risk of mortality [41]. The primary treatment of these aggressive variants of PMGCTs is surgery, as most of them are not sensitive to cisplatin-based chemotherapies. Inoperable cases may be treated with chemotherapy regimens chosen according to the somatic transformation type [42,43].

## 8. Diagnosis and Pre-Treatment Evaluation

GCTs are usually located in the anterior mediastinum and in differential diagnosis, thymic diseases, thyroid goiter, and lymphomas should be considered. PMGCT diagnosis and characterization may be challenging. In most cases, the diagnosis is based on small needle core biopsies and further immunohistochemistry (IHC) studies are often needed for confirmation. As non-diagnostic needle biopsies constitute a significant concern, wide tissue sampling and attentive diagnostic evaluation are essential [44,45]. Usually, patients are given cisplatin-based chemotherapy before surgery, which makes the first pathologic evaluation extremely important. Indeed, the existence of somatic differentiation, which indicates an unfavorable prognosis, should be confirmed before treating patients with chemotherapy, as chemotherapies induce tumor necrosis which could decrease the transformed component. Additionally, as β-HCG might be within the normal range or mildly elevated in both seminoma and non-seminoma GCTs, a biopsy is mandatory for disease identification. Conversely, the disease might not need to be biopsy-proven if the patient has high AFP measurements [46].

Routine CBC and blood biochemistry with AFP, β-HCG, and LDH should be obtained. In the case of clinical suspicion, testicular examination with ultrasonography is necessary. Orchiectomy should be performed for equivocal findings to rule out metastasis from a gonadal primary, as there could be no mass but rather a scar-looking lesion with the burned-out tumor context.

There is no officially recognized AJCC TNM staging protocol for extragonadal GCTs. Pre-treatment assessment with CT imaging/PET scanning to define the anatomy and vascular relationship and to examine the spread of the disease before surgery is essential. MRI is also a valuable modality to explore adjacent structure invasion. Seminomas present with large homogeneous soft tissue masses similar to lymphomas, whereas non-seminoma GCTs are inhomogeneous tumors with border irregularities owing to their invasive behavior. In radiological studies, teratomas appear as a round, multi-lobular inhomogeneous soft tissue mass with calcification (20–43%) [47]. Brain MRI and a bone scan can also be performed if the patient has suspicious symptoms of metastasis since they can metastasize to the brain and bones [48,49].

Patients should be counseled about semen analysis and sperm banking according to the patient’s desire to have children before starting chemotherapy as sperm count and quality might be affected by chemotherapy. In addition, infertility itself is a risk factor for the development of testicular cancer, and DNA repair defects might explain the correlation between impaired spermatogenesis and carcinogenesis [50]. Whether azoospermic men require cancer screening is an area that needs further investigation.

## 9. Treatment

The treatment goal for PMGCT patients is cure. Even in patients with extensive disease, treatment is curative in > 80% with seminoma PMGCTs and in 40–50% of patients with non-seminoma PMGCTs using a multimodality approach. Appropriate treatment should be initiated as soon as possible. Complete or near-complete surgical resection after normalized or decreased serum tumor markers has an important impact on the treatment of patients with PMGCTs. Parameters that are indicative of good prognosis after primary chemotherapy include complete resection, less than 10% viable tumor cells present in the resection material, and good prognosis classification according to IGCCCG [8,44,45,51]. Conversely, immature teratoma and non-seminoma subtypes indicate a worse prognosis.

Although non-seminoma PMGCTs have less sensitivity to platinum-based chemotherapies, seminoma and non-seminoma patients are most often treated with bleomycin, etoposide and cisplatin (BEP), or etoposide and cisplatin (EP), depending on the IGCCCG risk group. Treatment with VIP protocol (substitute ifosfamide for bleomycin) would be a better option over the standard BEP regimen for the possibility of future thoracic surgery. Bleomycin treatment constitutes a risk to both surgical and postoperative morbidity and mortality. The mechanism of bleomycin-induced lung injury is mainly related to oxidative damage in genetically susceptible patients, which presents with interstitial pneumonitis leading to fibrosis. The diffusing capacity of the lungs for carbon monoxide (DLCO) is an easy and accessible way of documenting subclinical bleomycin-induced lung toxicity. Treatment should be withheld if the diffusion capacity falls below 30–35% of the initial measurement, and doses should not exceed 400 units as this will increase the lung toxicity risk. The anesthesiologist should be informed about bleomycin exposure to take preventive measures, including using a low fraction of inspired oxygen and restricting fluid replacement during the operation.

For non-seminoma histology, four cycles of chemotherapy followed by post-chemotherapy surgical resection is recommended. Post-chemotherapy surgery is crucial as the residual tumor may contain viable residual germ cells, immature or mature, or teratoma with somatic differentiation which are related to poor prognosis. Rising post-chemotherapy tumor markers do not preclude successful therapy with surgical resection due to the poor response rates with salvage chemotherapy [52]. Treatment of relapsed disease is challenging given the restricted effectivity of standard or high-dose regimens. Radiation therapy has no role in treating primary non-seminoma GCTs but could be considered to manage specific conditions, such as for brain metastases.

A growing mediastinal mass associated with cardiopulmonary function deterioration is expected in the case of growing teratoma syndrome, a clinical condition that should be suspected when there is progression of the disease despite tumor markers declining on chemotherapy [53]. Early recognition of growing teratoma syndrome is important for proper management. In these cases, early surgical intervention is recommended and is associated with better outcomes [53,54]. An aggressive surgical procedure may also be required in chemotherapy-refractory PMGCTs.

Upfront surgery is an option for patients with resectable tumors and negative tumor markers. High-dose chemotherapy (HDCT) and peripheral blood stem cell transplantation (PBSCT) might be another treatment option for patients with persistently elevated postoperative serum tumor markers and recurrent non-seminoma PMGCT. However, it should be noted that having non-seminoma PMGCT is a negative prognostic factor, which indicates a poor outcome in patients treated with HDCT and PBSCT. Therefore, some experts do not prefer using HDCT in non-seminoma PMGCTs. On the other hand, a limited number of patients responded well to salvage HDCT and PBSCT [55,56]. As this patient group is excluded from most of the HDCT studies, more evidence is needed about HDCT and PBSCT to better select the patients who will respond to therapy. Following HDCT and PBSCT, surgery needs to be performed on residual masses with curative intent, possibly in large volume centers to improve outcomes [57].

The prognosis of seminoma PMGCTs patients is similar to gonadal seminoma patients with a survival rate exceeding 90% at 5 years. Extrapulmonary visceral metastases and metastases to two or more different sites are poor prognostic features [44]. PMGCTs without non-pulmonary metastases are categorized as good risk according to the IGCCC risk classification. The good prognosis of seminoma PMGCTs is dictated by their exquisite radiotherapy and chemotherapy sensitivity. Initial surgical excision with adjuvant chemotherapy is also an acceptable approach for small resectable tumors in asymptomatic patients. If the tumor is not resectable upfront, chemotherapy eventually followed by surgery or radiation therapy of the residual tumor is the preferred approach. In patients with good IGCCCG risk, three cycles of BEP (or four cycles of EP chemotherapy if bleomycin is contraindicated) is recommended [58]. In patients with moderate IGCCCG risk, four cycles of BEP or VIP are the recommended options. Although chemotherapy is the preferred treatment, radiation therapy to the mediastinum (35–50 Gray) could represent an option in patients with major contraindications to chemotherapy and without bulky disease.

Management of post-chemotherapy residual masses depends on the primary tumor histology and the size of the residual disease. In seminoma PMGCTs, residual masses <3 cm can be closely monitored with repeated CT scans. Masses >3 cm are more suspicious of harboring viable malignant disease and can either be closely monitored with serial CT scans or, in case of growing or stable lesions, by open biopsy. FDG PET scan could be valuable to rule out residual viable seminoma, if it is negative. However, the treatment decision should not rely on a positive PET scan as the positive predictive value of this test is low and this could lead to a significant risk of overtreatment. Residual viable disease requires chemotherapy or radiation therapy if surgery is not suitable. Post-chemotherapy progressing lesions require surgery if possible or salvage chemotherapy. A sternotomy, thoracotomy, VATS, or robotic surgical technique may be used to remove residual masses [59]. In patients with post-chemotherapy non-seminoma PMGCTs, surgery of residual masses is indicated if feasible. PET scans have a very limited utility in these patients because of the known lack of FDG uptake of teratomas [60,61].

Mature primary mediastinal teratomas can be cured with surgery alone with an excellent prognosis. Teratomas with somatic transformation are treated with surgery +/− chemotherapy according to the type and percentage of the transformed tumor.

Generally, serum tumor marker monitoring should be performed before each cycle of chemotherapy. Above all, center experience in poor prognosis patients’ treatment is associated with significantly better outcomes; therefore, referring patients to centers with expertise in managing GCTs should be considered.

## 10. Follow-Up and Survivorship

Patients are usually followed up every three months for the first two years and every six months for the next three years with CT chest and tumor markers. Patients should be monitored for acute and long-term treatment-related toxicities and recurrence. In the long run, patients may experience kidney dysfunction, hearing loss, neurotoxicity, infertility, and pulmonary diseases [62].

Nephrotoxicity is closely associated with the number of cycles. While >400 mg/m^2^ of cisplatin resulted in around a 20% decrease in GFR from the baseline after 5 years, kidney function returns to nearly normal in most patients [63]. Adequate hydration, maintaining normal blood pressure, and avoiding nephrotoxic agents may help with short- and long-term nephrotoxic effects.

Cisplatin-associated peripheral neuropathy was reported in 20–30% of patients as a long-term adverse effect. Increased age, smoking, excessive alcohol consumption, and hypertension were the variables related to increased toxicity [64]. Additionally, a total cisplatin dose above 300 mg/m^2^ is associated with an increased risk of neurotoxicity and ototoxicity. Preliminary data showing a relationship with genetic background need further validation [65]. Unfortunately, no proven preventive measures exist against these toxicities. Duloxetine may be used for neural symptom management as per the guidelines, and symptoms might improve partially with time [62]. At least one audiometric analysis should be carried out for ototoxicity in the post-chemotherapy period.

The incidence of hypogonadism is high in this patient population, increasing the risk of metabolic syndrome and psychological distress. Testosterone and LH should be ordered for Leydig cell assessment at any point in those with sexual dysfunction and testosterone replacement should be considered. Patients may have anxiety about the fear of cancer coming back and related sociologic problems about engaging in daily life [66]. Several tools are available to assess the individual’s mental state and quality of life. In the suspicion of poor mental health, patients should be counseled appropriately. Cognitive decline and brain fog might be experienced by some patients while on treatment or after treatment. Early detection and cognitive rehabilitation might be related to better outcomes [67].

The relative risk of having cardiovascular disease (CVD) for those treated with chemotherapy is between 1.4 and 7.1 [68]. Dying from cardiovascular disorders in patients with mediastinal GCTs increases four-fold compared to those with gonadal GCTs, which might be attributed to the utilization of more aggressive treatments. The increased incidence of CVD might be associated with cisplatin-related vascular endothelial damage and hypogonadism-related metabolic disorders [68,69]. It is essential to consider that a higher body mass index and an increased incidence of hyperlipidemia, diabetes, and hypertension will contribute to the risk of cardiovascular events. If an accompanying significant family history of cardiovascular disease is also present, healthcare providers should take extra care to encourage patients to adopt a healthy lifestyle, such as limiting alcohol, tobacco, and dietary fats and exercising regularly as measures against negative behaviors, which can reduce CVD by 80% [70]. In addition, cardiovascular monitoring is required at regular intervals according to the risk category.

There is also a risk of developing secondary malignancies due to chemotherapy and radiotherapy. Radiotherapy-induced secondary malignancy largely depends on the dose and field of the radiation treatment. While cisplatin was found to be associated with an increased risk of head and neck, esophageal, lung, and bladder cancers, radiation therapy was associated with head and neck, stomach, liver, pancreatic, and bladder cancers [71]. Cisplatin and etoposide may also cause secondary hematologic malignancies. Patients should be advised to monitor their symptoms and consult their healthcare providers if necessary.

While bleomycin can cause an increased risk of acute toxicity, chronic complications are not prevalent. Most patients’ pulmonary function returns to normal at 5 years, except those with a poor-risk disease, with them receiving multiple lines of treatment and being treated with pulmonary surgery [72]. Radiotherapy and pulmonary embolism could also be responsible for impaired pulmonary function. Smoking cessation should be encouraged, and bleomycin should be omitted in patients older than 40 years and those who have known lung and kidney diseases.

The mediastinal primary site has a greater risk of toxicity and non-cancer-related death due to the need for aggressive treatment. Therefore, it is important to encourage patients to report any new or persistent symptoms.

## 11. Conclusions

PMGCTs represent a heterogeneous entity with distinct clinical and molecular features between non-seminoma and seminoma MGCTs. Non-seminoma PMGCTs remain among the poorest prognostic group in the realm of GCTs because of their low sensitivity to chemotherapy and their high risk of relapse [73]. Integrated, multidisciplinary treatment is paramount to maximize cure rates in this patient population. Conversely, seminoma PMGCTs have an excellent prognosis that is comparable to their gonadal counterpart with a five-year overall survival rate exceeding 90% with the use of a multidisciplinary approach.

Identification of biological and genetic factors to predict treatment responses would be beneficial for implementing treatment strategies and would ultimately improve patients’ outcomes. Moreover, involving centers with expertise in the care of GCTs is associated with significantly better outcomes.

## Figures and Tables

**Table 1:** Survival updates on poor-risk GCTs.

	Years	Number of Patients	5-Year PFS (95% CI)	5-Year OS (95% CI)	Ref.
IGCCCG	1975–1990	832	41% (35 to 47)	48% (42 to 54)	[9]
Gillessen et al. (IGCCCG Update)	1990–2013	2514	54% (52 to 56)	67% (65 to 69)	[10]
Adra et al.	1990–2014	273	58% (51% to 63%)	73% (67% to 78%)	[11]

IGCCCG, International Germ Cell Cancer Collaborative Group; PFS, progression-free survival; OS, overall survival; CI, confidence interval.

**Table 2:** Immunohistochemistry in germ cell tumors.

	Seminoma	Immature Teratoma	Yolk Sac Tumor	Embryonal Carcinoma	Choriocarcinoma
Positive Markers	SALL4 OCT 3–4 NANOG c-kit PLAP	SALL4 SOX2 Cytokeratins EMA	SALL4 Glypican-3 Cytokeratins AFP	SALL4 OCT 3–4 CD30 SOX2 Cytokeratins NANOG	HCG Cytokeratins Glypican-3 SALL4 EMA
Negative Markers	CD30 Glypican-3 SOX2	OCT 3–4 CD30 c-kit NANOG SOX2	CD30 c-kit SOX2 NANOG OCT 3–4	c-kit Glypican-3	OCT 3–4 c-kit CD30 SOX2 NANOG

## Data Availability

No new data were created or analyzed in this study. Data sharing is not applicable to this article.
